# Endoscopic vs. microscopic tympanoplasty in children: a retrospective case-control study

**DOI:** 10.3389/fsurg.2025.1649552

**Published:** 2025-09-16

**Authors:** Raffael Fink, Sven Beckmann, Sean C. Sheppard, Marco Caversaccio, Lukas Anschuetz

**Affiliations:** ^1^ Department of Otorhinolaryngology, Head and Neck Surgery, Inselspital, Bern University Hospital, University of Bern, Bern, Switzerland; ^2^ Université Paris Cité, Institut Pasteur, AP-HP, Inserm, CNRS, Fondation Pour l’Audition, Institut de l’Audition, IHU ReConnect, Technologies et Thérapie Génique Pour la Surdité, Paris, France; ^3^ Department of Otorhinolaryngology, Head and Neck Surgery, CHUV, University of Lausanne, Lausanne, Switzerland; ^4^ The Sense Innovation and Research Center, Lausanne and Sion, Lausanne, Switzerland

**Keywords:** endoscopic ear surgery, microscopic ear surgery, pediatric, cholesteatoma, tympanoplasty, hearing loss

## Abstract

**Purpose:**

To compare the outcomes of endoscopic and microscopic tympanoplasty (TPL) types I-III in pediatric patients.

**Methods:**

A retrospective case-control study was conducted on 70 TPL cases in 58 pediatric patients at Inselspital, Bern University Hospital, Switzerland, from June 2017 to December 2023. Data on hearing function, graft intake, residual disease, operating time and complications were collected.

**Results:**

We observed mean postoperative air-bone gap (ABG) of 16.83 dB using the endoscopic and 19.37 dB for the microscopic techniques, as well as a higher graft intake rate (91%) for the endoscopic compared to the microscopic group (80%), although these differences did not reach statistical significance. No residual cholesteatoma was found in the endoscopic group, while the microscopic group had a significantly higher incidence of residual disease (42%; *p* = .03). The mean operative time was shorter in the endoscopic group (87 min vs. 113 min; *p* < .01). Postoperative complications were lower in the endoscopic group compared to a 14% incidence in the microscopic group.

**Conclusion:**

Endoscopic tympanoplasty in pediatric patients achieves similar audiological outcomes and graft intake rates compared to the microscopic approach while offering significant advantages, including reduced operation times and lower complication rates. This minimally invasive approach is highly effective especially regarding cholesteatoma resection and provides excellent functional and structural outcomes.

## Introduction

Children with chronic otitis media (COM) suffer from tympanic membrane (TM) perforation, which may be associated with ossicular chain disruption, leading to conductive hearing loss and more or less frequent superinfections. At a young age impaired hearing negatively affects language acquisition and learning development (1). Tympanoplasty (TPL) represents the surgical gold standard treatment aiming to remove the disease, repair the TM and restore ossicular chain integrity. While the operation was historically commonly approached with a microscope, the use of an endoscope has been increasingly reported in the last decades. Studies on surgical hearing restoration favor less invasive transcanal methods, which reduce the need for extensive bone drilling and postauricular incisions (2). These surgical approaches result in shorter hospital stays and faster return to physical activities (3).

While at birth the dimensions of the TM and middle ear are comparable to those of adults, the external auditory canal (EAC) is subject to significant growth approximately until the age of six (4, 5). The question arises, whether transcanal surgery and in particular endoscopic surgery is suited for the pediatric population. It has been observed, that the relatively short EAC in children permits wide-ranging maneuverability of the endoscope and instruments, compensating for its narrow diameter. Moreover, the wide-angle view from the tip of the endoscope provides a panoramic view of the middle ear recesses (6). The extent to which a microscope or an endoscope is used in pediatric ear surgery depends largely on the need of bone drilling, the ear canal condition, available resources, and surgeon expertise. With appropriate circumstances, a full range of otologic procedures such as cholesteatoma removal, all types of TPL, stapes surgery and treatment of skull base pathology can be performed partially or entirely using the endoscopic technique (7–10).

Comparative studies between endoscopic and microscopic techniques have reported similar auditory outcomes and graft intake rates. However, the endoscopic approach has shown potential benefits, such as reduced postoperative pain, faster recovery, and lower rates of residual cholesteatoma (11–14). At our center, both transcanal endoscopic and microscopic approaches are routinely employed. The aim of this retrospective case-control study is to compare the outcome of the two techniques in TPL type I-III according to the Wullstein classification (15) in pediatric patients.

## Materials and methods

Pediatric patients who underwent endoscopic or microscopic TPL type I-III with or without cholesteatoma at our tertiary referral center, in the period between June 2017 and December 2023 were retrospectively reviewed. A total of 70 consecutive ears involving 58 patients younger than 18 years old, who underwent Type I-III TPL were included. Exclusion criteria included lack of available pre- and postoperative audiometric data and less than 3 months of audiometric and clinical follow up (FU). Institutional review board approval was obtained at the institutional and regional review board (Kantonale Ethikkomission, KEK-BE 2019-00555), and a waiver of informed consent was granted for this retrospective study.

### Surgical techniques

A total of 35 consecutive endoscopic approaches were matched with consecutive microscopic cases. At our institution, the endoscopic technique was newly introduced during the study period. Surgeons who adopted the endoscopic approach consistently applied it to all cases, whereas those not yet familiar with the technique continued to utilize the microscopic approach. Thus, the choice of surgical method was not made on a per-case basis, but rather reflected a transition in institutional practice and individual surgeon expertise. This allowed for a direct comparison of outcomes between the two approaches within a comparable pediatric population. The surgical approach involved either the endoscopic method or the microscopic “inside-out” technique, eradicating the disease from the middle ear toward the mastoid (16). The use of the endoscope in the respective cases was classified by Cohen et al. (17) Cases rated as class 2b or higher were categorized within the endoscopic group, including cases requiring mastoidectomy due to disease extension beyond the lateral semicircular canal into the mastoid. In these cases, after mastoidectomy, the reconstruction of the middle ear was conducted endoscopically (Cohen Class 2b). In all cases, the defect of the posterior canal wall end epitympanum was reconstructed with cartilage. Only one case requiring canal wall down mastoidectomy underwent obliteration of the cavity using a pedicled random flap. Matching was based on surgical indication, type of ossicular chain reconstruction, and age. All type I TPL were performed in underlay technique. If a cartilaginous graft was used, the perichondrium was removed from the concave surface while preserved on the convex side, and the graft was positioned with the perichondral surface facing laterally. Incus interposition, Titanium partial ossicular replacement prostheses (PORP) and total ossicular replacement prosthesis (TORP), cartilage interposition, and bone cement ossiculoplasty (OPL) were included as methods of OPL.

### Data collection

Data regarding pre- and postoperative hearing function, status of the TM and the ossicular chain, presence and extent of the cholesteatoma according to EAONO-JOS classification (18) or the underlying disease, surgical procedure, type of graft or prosthesis used, and perioperative complications were retrospectively collected from patients' charts. Where necessary, the surgical videos were reviewed. Postoperative assessment was carried out 3 months after surgery. Bone conduction (BC) and air conduction (AC) pure-tone average (PTA) were calculated, from pre- and postoperative pure-tone audiometry, as the mean value among thresholds at 0.25, 0.5, 1, 2, and 4 kHz frequencies. Mean air–bone gap (ABG) was calculated as the difference between AC-PTA and BC-PTA, while ABG improvement was calculated as the difference between mean pre- and mean postoperative ABG, with a positive value indicating an improvement and a negative value indicating a worse postoperative gap. All cases of cholesteatoma underwent primary OPL and received radiological FU with diffusion-weighted MRI one year after the surgery. Residual cholesteatoma was defined as cholesteatoma behind an intact neotympanum. In contrast, recurrent cholesteatoma is the appearance of a recurrent TM retraction and cholesteatoma.

### Statistical analysis

Continuous variables such as audiometric outcomes and operative times were compared between the endoscopic and microscopic techniques using unpaired t-tests, whereas paired t-tests were applied within each group to compare pre- and postoperative audiometric outcomes. Categorical variables including graft intake and complications were compared using Fisher's exact test.

Statistical significance was set at *p* < .05. Results are presented as mean ± standard deviation for continuous variables and as proportions for categorical variables. Statistical analyses were performed with GraphPad Prism 10.0 (GraphPad, La Jolla, CA).

## Results

In this retrospective case-control study we matched a total of 70 consecutive endoscopic and microscopic TPL type I–III. The patients' age ranged from 4 to 17 with a mean age of 12.89 years (SD ± 3.58) in the endoscopic and 12.40 years (±3.56) in the microscopic group. The patient's characteristics are summarized in Table 1. COM was the primary surgical indication in both groups, with 16 cases in the endoscopic and 15 in the microscopic group. Cholesteatoma was found in an additional 12 cases per group. Other indications included conductive hearing loss due to atelectasis or malformation of the ossicular chain with intact footplate mobility. Nine endoscopic and six microscopic cases were revision surgeries due to persistent or recurrent disease.

**Table 1:** Description of patients, surgical therapy, and outcome.

Characteristics	Endoscopic (*n* = 35)	Microscopic (*n* = 35)	*P*
Sex			
Female	12	14	
Male	23	21	
Age, mean (± SD)	12.89 (±3.58)	12.40 (±3.56)	
Ear (right)	12	13	
Revision surgery	9	6	
Surgical indication			
Chronic otitis media	16	15	
Atelectasis	4	5	
Malformation	3	3	
Cholesteatoma	12	12	
Cholesteatoma stage			
I	1	2	
II (M+)	11 (4)	10 (5)	
Intervention			
TPL Type I	12	12	
Chole	12	12	
TPL Type II-III	11	11	
Transcanal exclusive	33	19	.001
CWU Mastoidectomy (CWD)	2	12 (1)	.003
Operation time, mean (± SD) minutes	87 (±41)	113 (±60)	.039
TPL Type I	53 (±12)	67 (±25)	
Chole	131 (±33)	175 (±48)	.019
TPL Type II-III	76 (±21)	96 (±38)	
Type II			
Bone cement	3	1	
PORP			
Incus interposition	5	7	
Titanium	4	4	
Cartilage	1		
TORP			
Titanium TORP	9	11	
Incus interposition	1		
Postoperative complications			.05
Wound infection	0	4	
Wound seroma	0	1	
Cholesteatoma			
Residual	0	5	.03
Recurrence	0	0	
Follow up months (± SD)	13.91 (11.28)	10.66 (10.41)	

Cholesteatoma staging according to EAONO/JOS Staging System; M+ indicates Mastoid involvement; *P* values are only indicated if *p* < .05; Postoperative complications defined as the first three months of follow up; Chole, cholesteatoma resection with tympanoplasty type II-III; CWD, canal wall down; CWU, canal wall up; PORP, partial ossicular replacement prosthesis; SD, standard deviation; TM, tympanic membrane; TORP, total ossicular replacement prosthesis; TPL, tympanoplasty.

**Figure 1:**
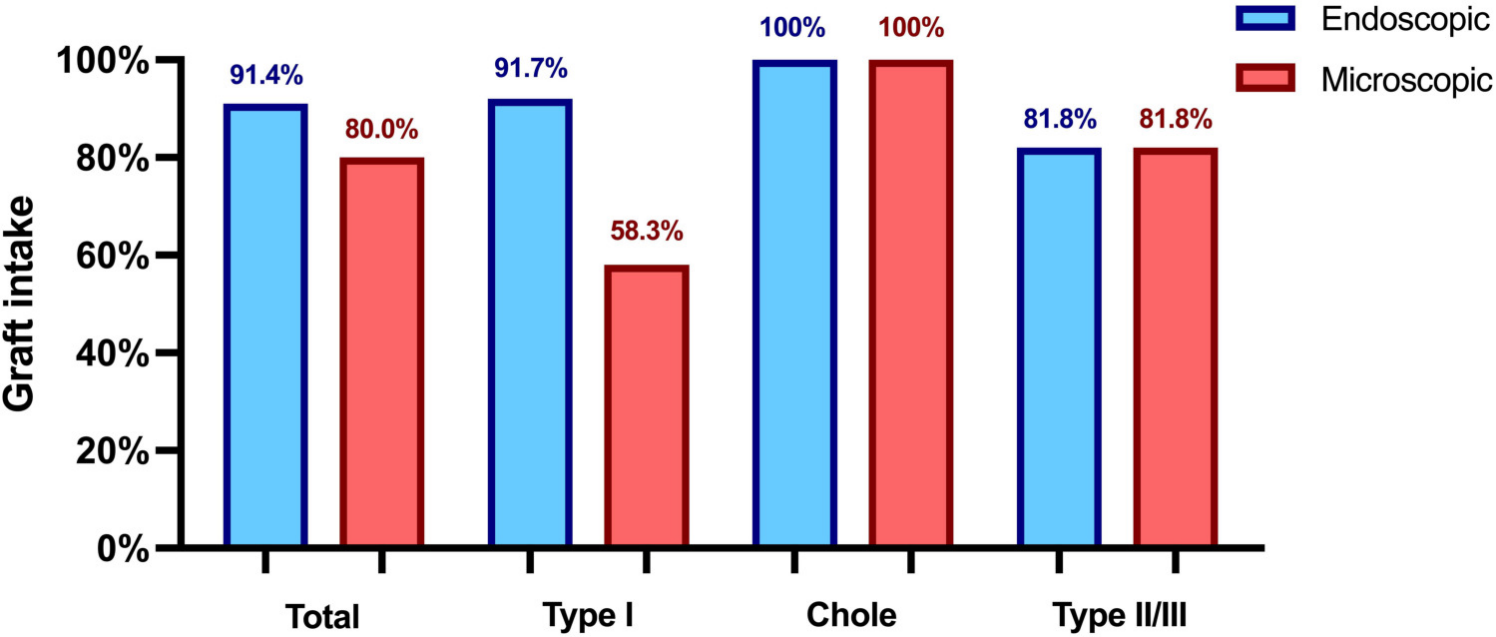
Graft intake rate 3 months postoperatively. Chole, cholesteatoma resection with tympanoplasty type II or III; Type I, tympanoplasty type I; Type II/III, tympanoplasty type II or III.

In type I TPL the reconstruction material in the endoscopic group included cartilage (*n* = 8), temporalis fascia (*n* = 3), and artificial membrane (*n* = 1), achieving a graft intake rate of 91.66%. In contrast, the microscopic group used fascia (*n* = 8) and cartilage (*n* = 4), with a lower graft intake rate of 58.33% (*p* = .16). A total of 46 ossicular chain reconstructions were conducted, including 13 PORP and 10 TORP in the endoscopic group, and 12 PORP and 11 TORP in the microscopic group, utilizing various reconstruction materials. Both techniques showed comparable graft intake rates, as indicated in Figure 1, with one postoperative prosthesis dislocation in the microscopic group.

**Figure 2:**
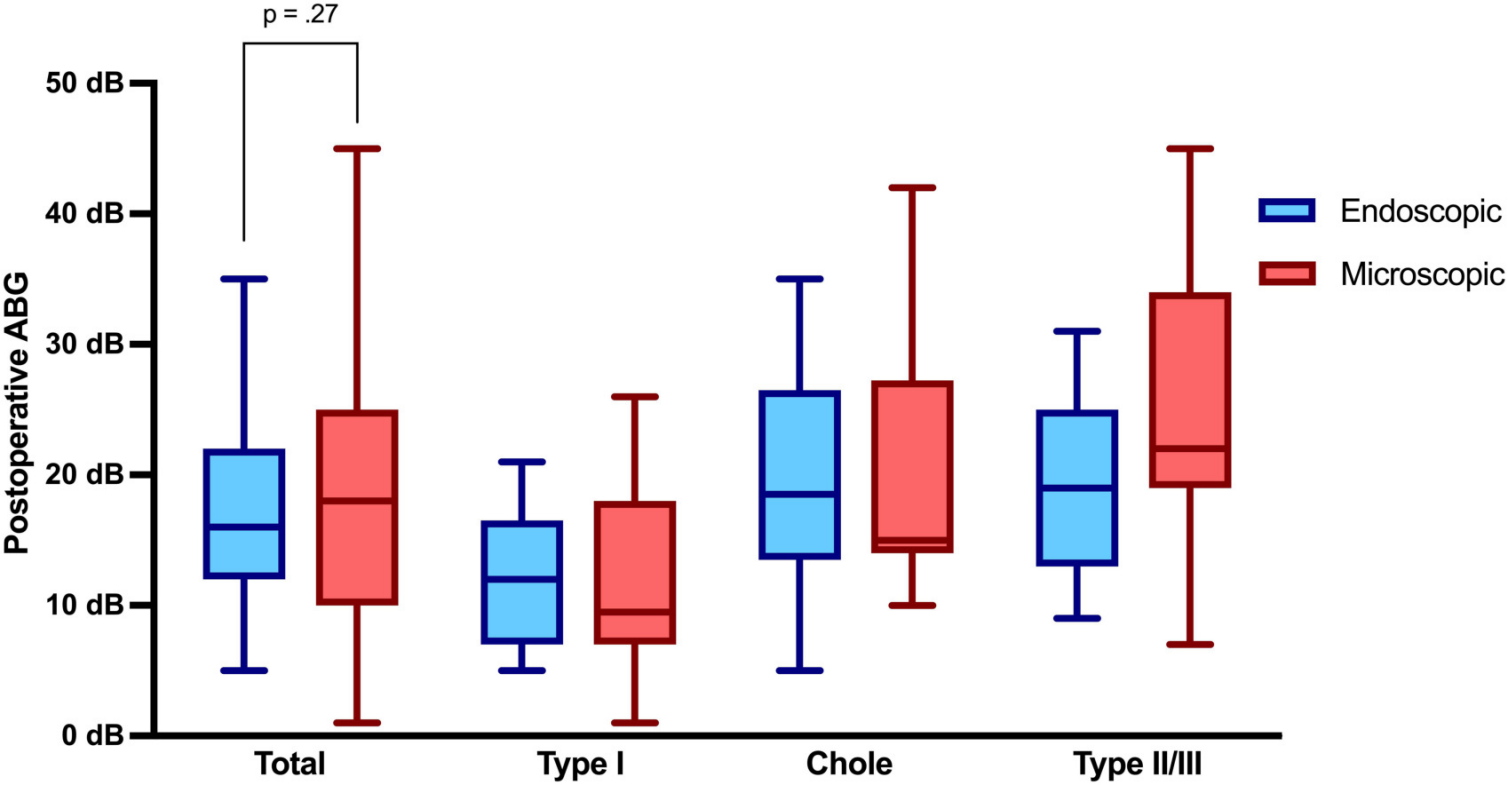
Air-bone gap 3 months postoperatively. ABG, air-bone gap; Chole, cholesteatoma resection with tympanoplasty Type II or III; Type I, tympanoplasty type I; Type II/III, tympanoplasty type II or III.

Audiological assessment revealed a statistically significant higher ABG improvement in the endoscopic group, with an average improvement of 14.7 dB compared to 7.9 dB in the microscopic group (*p* = .02). Preoperative ABG was higher in the endoscopic cohort with 31.5 dB (±12.13), compared to 26.4 dB (±12.10) not reaching statistical significance (*p* = .09). Tables 2a, 2b illustrate pre- and postoperative ABG according to the two techniques. As depicted in Figure 2, the postoperative ABG values were comparable between the endoscopic 16.83 dB (±7.74) and microscopic 19.37 dB (±11.01) approaches (*p* = .27).

**Table 2a:** Endoscopic technique: breakdown of postoperative air-bone gap of 20 dB or lower according to the preoperative hearing loss.

Air-bone gap	Preoperative				
Postoperative	<10 dB	11–20 dB	21–30 dB	31–40 dB	>40 dB
	*n* = 2	*n* = 4	*n* = 11	*n* = 9	*n* = 9
<10 dB	50.0	50.0	27.3	11.1	
11–20 dB	50.0	50.0	36.4	66.7	55.6
21–30 dB			36.4	11.1	33.3
31–40 dB				11.1	11.1
>40 dB					
Total <20 dB	100.0	100.0	63.6	77.8	55.6

Values are expressed in percentages.

**Table 2b:** Microscopic technique: breakdown of postoperative air-bone gap of 20 dB or lower according to the preoperative hearing loss.

Air-bone gap	Preoperative				
Postoperative	<10 dB	11–20 dB	21–30 dB	31–40 dB	>40 dB
	*n* = 3	*n* = 7	*n* = 17	*n* = 4	*n* = 4
<10 dB	100.0	14.3	23.5		25.0
11–20 dB		14.3	47.1	75.0	25.0
21–30 dB		57.1	17.6		25.0
31–40 dB			5.9	25.0	
>40 dB		14.3	5.9		25.0
Total <20 dB	100.0	28.6	70.6	75.0	50.0

Values are expressed in percentages.

The overall mean operation time was statistically significantly shorter in the endoscopic group with 87 min (± 41) compared to 113 min (±60) in the microscopic group (*p* = .04), particularly during cholesteatoma resection. Exclusive transcanal surgery was performed in 33 of the 35 endoscopic cases, while two cases needed mastoidectomy due to disease extension (Cohen 2b). In contrast, 16 microscopic cases required a retroauricular approach, with a statistically significant increased number of mastoidectomies performed (*p* = .03). Postoperative FU revealed higher complication rates in cases requiring a retroauricular approach, with four incidents of wound infection and one seroma, necessitating surgical revision (*p* = .05). In our cohort, the mean FU period was 13.91 months (±11.28) for the endoscopic group and 10.66 months (±10.41) for the microscopic group. Specifically, among cholesteatoma cases, the mean FU period was longer with 18.58 months (±12.70) for the endoscopic group and 15.17 months (±10.45) for the microscopic group. Residual cholesteatoma was significantly more frequent in the microscopic group (*p* = .03), while no recurrences were reported in either group.

## Discussion

This retrospective case-control study evaluates the outcomes of endoscopic vs. microscopic techniques in pediatric TPL types I-III. The two approaches showed comparable postoperative ABG values, while the endoscopic approach showed significantly higher ABG closure and shorter operating times. Additionally, it allowed for more frequent total transcanal surgeries, minimizing the need for retroauricular approaches, while reducing the incidence of residual cholesteatoma and postoperative complications.

### Audiological outcome and graft intake

Previous studies have reported similar audiological outcomes between endoscopic and microscopic techniques in pediatric ear surgeries (12, 14, 19). Consistent with these findings, postoperative ABG values in our cohort were comparable between the two techniques. However, ABG closure was significantly increased (*p* = .02) in the endoscopic group, particularly in type II and III TPL without cholesteatoma. The enhanced visualization provided by endoscopy, which offers better illumination of anatomical details of the middle ear compared to an operating microscope, may contribute to more accurate prosthesis placement and, consequently, improved hearing outcomes. Another factor in the described cohort was the higher preoperative ABG in the endoscopic group facilitating an increased ABG closure. Among the type I TPL and cholesteatoma cases, ABG closure was comparable between the endoscopic and microscopic techniques. Enhanced graft placement in the endoscopic technique might also contribute to the higher graft intake rates observed in our type I TPL cohort (92% vs. 58%), although this difference did not reach statistical significance due to the cohort size. Moreover, the choice of graft material constitutes a relevant confounding factor, limiting the extent to which the observed differences in graft success can be attributed to the surgical approach alone. Previous studies have reported comparable hearing outcomes and graft intake rates for both endoscopic and microscopic type I TPL (20, 21). Therefore, the discrepancy in graft intake observed in our study may be related to differences in graft selection, with cartilage predominantly used in the endoscopic group and fascia in the microscopic group. This aligns with findings from other studies, which have reported differences in graft intake rates between tragal cartilage and fascia (22, 23). The superior visualization provided by the endoscopic technique, combined with the prevalent use of cartilage, may have contributed to the higher graft intake rates observed, while maintaining excellent audiological outcomes comparable to those achieved with fascia grafts.

### Transcanal surgery

The limiting factors for transcanal surgery in children are the curvature and the narrow width of the bony meatus, which complicates especially microscopic inspection of the TM and middle ear. The endoscopic technique, with its varying diameters of rigid endoscopes, is less hindered by the small width. Moreover, the use of angled endoscopes allows complete examination of the hidden recesses of the middle ear, except in the rare occurrence of a type C retrotympanic recess (posterior and medial to the facial nerve) (24). Caloway et al. reported a significantly lower need for a postauricular approach with the endoscopic technique (19). These findings are supported by our study, showing a significantly higher frequency of postauricular approaches in the microscopic technique (*p* < .01). In our cohort, the smaller pediatric ear canal did not impede endoscopic transcanal access from the age of 4 years and for any type of TPL. Additionally, the thorough inspection of the middle ear allowed transcanal endoscopic cholesteatoma removal in most cases (83%). The two endoscopic cases that required a postauricular incision necessitated mastoidectomy due to disease spread into the mastoid. Our experience shows that depending on the specific anatomy of the middle ear and mastoid, cholesteatoma extending up to the dome of the lateral semicircular canal can be efficiently treated through an exclusive endoscopic approach. In cases of cholesteatoma extension beyond the lateral semicircular canal, managing the middle ear by the endoscope and supplement the resection by canal wall up mastoidectomy is reasonable as also proposed by James et al. (5) Given that radiological assessments frequently overestimate the extent of cholesteatoma in various middle ear subspaces compared to intraoperative findings, initiating surgery with a transcanal endoscopic approach appears to be a feasible strategy in order to minimize the needs for retroauricular incisions (25).

### Residual cholesteatoma

Surgical preservation of the mastoid air cell systems is thought to play an important role in maintaining pressure equilibrium within the middle ear by acting as a pressure buffer. Transcanal access with mastoid preservation may help prevent recurrences by maintaining middle ear gas homeostasis. Presutti et al. reported a significant reduction in recurrence rates for primary acquired attic cholesteatoma using transcanal endoscopic approach with mastoid preservation (26). According to James et al. young age, cholesteatoma extension and mastoid involvement increase the risk of recurrence in children. Surgical approach did not have a significant effect on this outcome (27). During the clinical and radiological FU in our cohort, no cases of recurrent disease were observed in both the endoscopic and the microscopic group, which is mainly due to the limited FU time. Our findings therefore refer exclusively to residual cholesteatoma. The microscopic group demonstrated a significantly higher incidence of residual cholesteatoma (*p* = .03), as depicted on FU MRI scans, and subsequently confirmed intraoperatively during revision surgery. The endoscopic technique showed no residual disease, whereas the microscopic technique showed residual disease in 42%. Simon et al. reported a 39% incidence of residual disease in children three years following a microscopic approach that did not routinely include endoscopic control (28). The enhanced visualization of the middle ear and the availability of digital enhancement technologies in endoscopic ear surgery facilitate complete disease elimination (29). A meta-analysis by Han et al. reported a significantly lower rate of residual disease with the endoscopic technique (*p* < .01) among children (14). Additionally, two authors reported a reduction in the risk of residual disease when the endoscope was used for dissection rather than just inspection (30, 31).

### Postoperative complications

Sparing retroauricular incisions and mastoidectomy in children not only reduces operating time and postoperative pain but also appears to lower the incidence of postoperative complications (13, 32). In their systematic review including both pediatric and adult population, Gkrinia et al. reported a significantly higher incidence of wound infections in the microscopic group, at 5%, compared to the endoscopic group, which had an incidence of 1% (33). Our results showed a 0% incidence of wound complications in the endoscopic cohort, whereas the microscopic cohort had an incidence of 14%. Notably, all wound infections including one seroma occurred following a retroauricular approach. Among the 18 retroauricular approaches, the incidence of wound complications reached 28%. Given the considerable complication rate associated with retroauricular approaches, we recommend whenever possible total transcanal surgery using the endoscope, as it significantly reduces the need for extending to a retroauricular approach.

### Limitations

This study has several limitations. The retrospective design and lack of randomization introduce a potential selection bias, as the choice of surgical technique was based on surgeon experience during a period of institutional transition. While matching was performed to improve comparability, residual confounding cannot be excluded. Additionally, the limited sample size may reduce the statistical power to detect more subtle differences between groups.

The relatively short FU period represents a major limitation, particularly in assessing long-term outcomes such as cholesteatoma recurrence, graft stability, and sustained hearing improvement. Although this reflects common challenges in pediatric otology FU, especially in asymptomatic children, it limits the ability to draw definitive conclusions on late outcomes. Prospective studies with extended follow-up are warranted to confirm these findings.

### Conclusion

Endoscopic ear surgery is highly effective for pediatric otology. This analysis showed reduced operating times and complication rates with comparable graft intake and audiological outcome in the endoscopic group. This minimally invasive technique offering unprecedented visualization, leads to excellent functional and structural outcomes, making it an optimal choice for pediatric patients.

## Data Availability

The raw data supporting the conclusions of this article will be made available by the authors, without undue reservation.
